# Associations between social norms and at-risk status for e-cigarette use: A sex-stratified analysis of Texas sixth-grade students

**DOI:** 10.1016/j.dadr.2024.100296

**Published:** 2024-11-09

**Authors:** Sarina A. Attri, Andrew E. Springer, Baojiang Chen, Steven H. Kelder, Dale S. Mantey

**Affiliations:** a The University of Texas at Austin, 110 Inner Campus Drive, Austin, TX 78712, USA; b Rollins School of Public Health, Emory University, 1518 Clifton Rd NE, Atlanta, GA 30322, USA; c Michael & Susan Dell Center for Healthy Living, Austin, TX 78701, USA; d University of Texas Health Science Center at Houston (UTHealth) School of Public Health, 1836 San Jacinto Blvd, Austin, TX 78701, USA

**Keywords:** E-cigarette, Adolescents, Social norms, Sex differences, Susceptibility

## Abstract

**Background:**

E-cigarette use remains high among adolescents, underscoring the need to identify targetable risk factors for intervention. This study examines associations between two social norms constructs (prevalence misperceptions and social acceptability) and at-risk status for e-cigarette use among Texas early adolescents.

**Methods:**

We conducted a cross-sectional analysis of baseline data from the CATCH My Breath study, which included n=1032 Texas sixth graders. Students who had ever used or were susceptible to using e-cigarettes were categorized as at-risk for long-term use. Susceptibility was measured using a 3-item index assessing curiosity, intentions, and receptivity to using e-cigarettes. Multi-level logistic regressions assessed associations between social norm constructs and at-risk status for the full and sex-stratified samples. Covariates were race, ethnicity, academics, household/peer tobacco use.

**Results:**

Overall, 36 % of 6th grade students were at-risk for e-cigarette use. Approximately 49 % of students overestimated peer e-cigarette use (“prevalence misperceptions”), and 43 % believed adolescent e-cigarette use is highly acceptable (“social acceptability”). Controlling for covariates, students with medium (aOR=1.89; 95 %CI=1.35–2.65) and high (aOR=1.98; 95 %CI=1.41–2.78) prevalence misperceptions had greater odds of being at-risk for e-cigarette use than those with low misperceptions. Students reporting medium (aOR=2.50; 95 %CI=1.66–3.76) and high (aOR=4.70; 95 %CI=3.21–6.90) social acceptability had greater odds of being at-risk for e-cigarette use than those reporting low acceptability. This association was stronger for females, relative to males.

**Conclusions:**

Greater prevalence misperceptions and social acceptability were associated with being at-risk for e-cigarette use among this sample of Texas early adolescents. Interventions should consider incorporating these social norms into intervention content.

## Highlights

• About half of students overestimated the prevalence of e-cigarette use.
• Nearly 3 in 4 students believed e-cigarette use is socially acceptable.
• Overestimating prevalence was linked to 1.98 times higher odds of e-cigarette risk.
• High social acceptability was linked to 4.71 times higher odds of e-cigarette risk.
• Social acceptability may be more strongly linked to e-cigarette risk for females.

## 1 Introduction

E-cigarette use has become increasingly prevalent across the world, especially in Europe and the United States (US) (eClinicalMedicine, 2022). Among US adolescents, approximately 10.0 % of high school students and 4.6 % of middle school students reported past 30-day (current) e-cigarette use in 2023 (Birdsey et al., 2023). The overall prevalence of adolescent e-cigarette use has decreased in the US since its historical peak in 2019. However, US middle school e-cigarette use is still increasing. About 550,000 middle school students were current users in 2023, up from 380,000 in 2022 (Birdsey et al., 2023, Park-Lee et al., 2022).

The high number of early adolescent e-cigarette users in the US is concerning given the adverse health effects for this age group. Risks of youth e-cigarette use include exposure to dangerous chemicals that can lead to chronic and acute lung disease, cardiovascular disease, addiction, and neurological deficits in the developing brain (Cao et al., 2020, Case et al., 2018, Eltorai et al., 2019, Hamberger and Halpern-Felsher, 2020, Livingston et al., 2022). These risks have led the United States Surgeon General (CDCTobaccoFree 2017CDCTobaccoFree, 2017; Surgeon General’s Advisory on E-cigarette Use Among Youth, 2023) and American Lung Association (American Lung Association, 2024) to make urgent calls to action to prevent e-cigarette use among young people.

Social norms are generally operationalized as a collective awareness of behaviors that are preferred and appropriate among a group of people (Chung and Rimal, 2016). Social norms are an ideal intervention target due to the relative ease of addressing people’s perceptions compared to other risk factors such as peer e-cigarette use, access to e-cigarettes, and exposure to advertising (Prentice, 2018). Additionally, there is substantial evidence that social norms predict initiation of traditional tobacco use behaviors among youth, which suggests that social norms may play a similar role in the initiation of e-cigarettes (Lahiri et al., 2024).

Social Cognitive Theory (SCT) provides a theoretical foundation for the role of social norms in influencing e-cigarette use. According to SCT, behavior is influenced by environmental factors such as normative beliefs, which are a type of social norm. Normative beliefs include an individual’s perceptions about how common a given behavior is among a group of people *(perceived prevalence)* as well as the perceived social acceptability of a given behavior *(social acceptability)* (Mantey et al., 2024). Prevalence misperceptions occur when individuals hold the false perception that a behavior is more prevalent than it is (Perkins et al., 2019), while social acceptability refers to perceptions about whether important people would approve of a behavior (Adkison et al., 2016, Primack et al., 2007). SCT posits that people are more motivated to engage in a behavior they believe is common or approved by role models or both (Mantey et al., 2024).

Despite a large body of research that has found social norms to be predictive of tobacco use among adolescents (Lahiri et al., 2024, Mantey et al., 2021), research is limited on the role of perceived social norms in e-cigarette use among adolescents (Agaku et al., 2020, Barrington-Trimis et al., 2019, Barrington-Trimis et al., 2018, Bold et al., 2018, Kreitzberg et al., 2018, Noland et al., 2016, Pepper et al., 2017, Pierce et al., 1996, Smith et al., 2021). For prevalence misperceptions and social acceptability in particular, past studies often measured these constructs as an outcome rather than risk factor, used solely qualitative data, or assessed associations with a more restrictive variable such as “vape tricks” (Agaku et al., 2020, Kreitzberg et al., 2018, Noland et al., 2016, Pepper et al., 2017, Smith et al., 2021). In guiding intervention development, we need to understand their relationships with both susceptibility and ever use, given that the US Food and Drug Administration (FDA) recommends using these variables to identify and target “at-risk” adolescents in prevention efforts (Santiago et al., 2019). Understanding how at-risk youth differ from their not-at-risk counterparts in their perceptions of social norms will allow for targeted interventions that prevent students from progressing to long-term e-cigarette use.

A growing body of evidence points to social norms as a predictor of adolescent e-cigarette initiation and potential intervention target (Agaku et al., 2019, Carey et al., 2019, Doxbeck and Osberg, 2021, East et al., 2019, Jiang et al., 2019, Noland et al., 2016, Rath et al., 2021, Yong et al., 2023). However, research is limited on the association between perceived social norms and e-cigarette use among early adolescents. Some prior studies did assess e-cigarette use as an outcome but focused on adolescents older than middle school age (East et al., 2019, Jiang et al., 2019, Kreitzberg et al., 2018, Pepper et al., 2017). Studies on younger adolescents are needed because e-cigarette use is increasing among middle school students, with the average age of initiation being below 14 years (Birdsey et al., 2023, Glantz et al., 2022, Park-Lee et al., 2022). Two studies assessed the relationship between prevalence misperceptions and e-cigarette use/susceptibility but used data from 2015. This was before the 2019 peak of the “vaping epidemic,” a term used to describe the alarming rates of youth e-cigarette use in the United States (Carey et al., 2019, Agaku et al., 2019, Jones and Salzman, 2020). Since 2015, popular e-cigarette brands have changed and marketing of flavors has increased, which may have affected adolescents’ perceptions of the prevalence and acceptability of e-cigarette use (Jones and Salzman, 2020). Furthermore, to the best of the authors’ knowledge, no studies have explored potential sex differences in the associations between social norms and use behaviors. As motivations for using e-cigarettes have been shown to vary by sex (Alam and Silveyra, 2023, Bedi et al., 2022), this is an important consideration in the development of prevention efforts.

In contributing to the limited body of research on the role of perceived social norms in e-cigarette use among early adolescents, this study examined the associations between two social norm constructs, *prevalence misperceptions of e-cigarette use* and *perceived social acceptability of e-cigarette use*, and at-risk status for e-cigarette use among a sample of Texas public middle school students. In addition, we explore possible sex differences in these associations. Given that limited research has investigated the association between social norms and e-cigarette use among early adolescence (i.e., 11–12 years of age), differences by sex, and among highly diverse samples, our study will contribute to addressing this gap.

## 2 Materials and methods

### 2.1 Study design and procedures

We analyzed baseline data from a randomized controlled trial of the CATCH My Breath adolescent e-cigarette prevention program. This study was a secondary cross-sectional analysis that did not factor in treatment condition, as we collected data before implementing the intervention. Participants were sixth-grade students recruited from n=22 public middle schools in the Dallas/Fort Worth and El Paso areas (Texas, United States).

Baseline data were collected via self-report surveys on Qualtrics from February to March 2021. A small subsample of students (n=71) completed an abridged survey on paper due to issues related to COVID-19. However, these observations were not included in the final sample, as the abridged version did not assess intermediate variables such as social norms. This study was approved by the University of Texas Health Science Center Committee for the Protection of Human Subjects [grant number HSC-SPH-18–0254] in April 2020. Parental/guardian consent was received for each student, and student participation was voluntary. Students who chose to complete the baseline survey were compensated with a $5 Amazon gift card.

### 2.2 Study sample

A total of n=1032 6th-grade students (predominantly aged 11–12) were included in the final analytic sample. While n=1244 students completed the initial survey, observations with missing data (n=141) for any of the variables of interest were excluded. There were no significant differences between the complete and incomplete cases for any of the measures of interest.

### 2.3 Measures

#### 2.3.1 At-risk for e-cigarette use

The outcome variable for this study was at-risk status for e-cigarette use. Participants were classified as at-risk for becoming regular e-cigarette users if they were susceptible never users or experimental ever users. Susceptibility indicates that a person is open to using e-cigarettes but has never tried one. Ever use indicates that a person has initiated e-cigarette use or experimented with an e-cigarette but is not a committed user. Research has shown that both susceptibility and ever use can lead to becoming a committed, regular user (Barrington-Trimis et al., 2019, Barrington-Trimis et al., 2018, Bold et al., 2018, Pierce et al., 1996). Classifying these two groups as at-risk for long-term use follows the FDA’s classification of youth at-risk for cigarette smoking and has been used by recent studies for e-cigarette use (Mantey et al., 2023, Santiago et al., 2019).

Participants were asked, “Have you ever used an electronic cigarette, even once? This includes JUUL, vape pens, mods, or any other type of e-cigarette.” Those who answered “yes” were categorized as ever-users and coded as at-risk for becoming a regular e-cigarette user in the future. Those who answered “no” were then assessed for e-cigarette susceptibility by being asked (1) “Have you ever been curious about using an e-cigarette?”; (2) “Do you think you will try an e-cigarette soon?”; and (3) “If one of your best friends were to offer you an e-cigarette, would you use it?”, with the possible response options “definitely yes,” “probably yes,” “probably not,” “definitely not.” Based on a validated approach for assessing e-cigarette susceptibility (Barrington-Trimis et al., 2019, Barrington-Trimis et al., 2018, Bold et al., 2018, Pierce et al., 1996), students were classified as susceptible to e-cigarette use if they answered with anything other than “definitely not” to at least one of the susceptibility questions. Those who were susceptible were included in the at-risk group, and those who were not susceptible were included in the not-at-risk group (referent group).

#### 2.3.2 Prevalence misperceptions

The prevalence misperceptions construct is used to assess the extent to which people’s perceived prevalence of e-cigarette use differs from its actual prevalence (Perkins et al., 2019). Similar to the pilot study of the CATCH My Breath program (Kelder et al., 2021, Kelder et al., 2020), students were asked how much they agreed with the following statements: (1) “Most students in my middle school use e-cigarettes”; (2) “Most students my age use e-cigarettes”; and (3) “Most kids in high school use e-cigarettes.” Responses ranged from 1 (strongly disagree) to 4 (strongly agree), and the values for each question were summed to create a prevalence misperceptions score. The total scores for the questions were split into tertiles to assign each student with low (scores 3–6), medium (score 7), or high (scores 8–12) prevalence misperceptions. Students in the medium and high categories at least somewhat agreed with one or more of the statements despite them each being false. Cronbach’s alpha for this variable was 0.77 using this study.

#### 2.3.3 Social acceptability

Social acceptability is a measure of normative beliefs that assesses how important it is to different people that the student does not use e-cigarettes. Based on previous studies (Adkison et al., 2016, Primack et al., 2007), the scale asked four questions, with answers ranging from 1 (strongly disagree) to 4 (strongly agree): (1) “How important is it to my parents that I do not use e-cigarettes?”; (2) “How important is it to my friends that I do not use e-cigarettes?”; (3) “How important is it to most people my age that I do not use e-cigarettes?”; (4) “How important is it to my teachers that I do not use e-cigarettes?” Similar to the prevalence misperceptions variable, responses to the four questions were summed to obtain a total score and tertiles were created due to a skew in the data to assign each student with low (score 16), medium (scores 14–15), or high (scores 4–13) social acceptability. Cronbach’s alpha for this study variable was 0.72; this was slightly lower than the alpha scores for items specific to cigarette smoking acceptability (0.85) (Adkison et al., 2016, Primack et al., 2007).

#### 2.3.4 Covariates

Several student-level covariates were included. Sex was a binary variable, with females as the referent group. Race and ethnicity were combined into a single variable, with categories including non-Hispanic white (referent), Hispanic/Latino, non-Hispanic Black, and non-Hispanic “Other” (i.e., American Indian or Alaska Native, Asian, Native Hawaiian or Other Pacific Islander, multi-racial, or any other race). Academic achievement was assessed by students’ responses to the question, “On average, what grades did you get in school last semester?” Possible categories were “Mostly As” (referent), “Mostly Bs,” and “Mostly Cs-Ds.”

Household e-cigarette or cigarette use was assessed with two separate questions that asked if any of the listed people in their household use e-cigarettes or smoke cigarettes. Students who indicated that at least one person in their household smokes or uses e-cigarettes were coded as 1 (i.e., “yes,” or positive for household use). Friend e-cigarette or cigarette use was similarly assessed, with two questions that asked how many of their close friends use e-cigarettes or smoke cigarettes. Those who selected anything other than “none of them” for either question were coded as 1 (i.e., “yes,” or positive for friend use).

### 2.4 Statistical analysis

We described the sample with frequencies and percentages for the total sample and based on at-risk status, prevalence misperceptions, and social acceptability. The percentage of students in each school who were economically disadvantaged, or eligible for the national free or reduced-price lunch program, was described using state-level data. Chi-square tests of independence (χ^2^) with corresponding degrees of freedom [χ^2^_(df)_] were computed to assess differences in sample characteristics based on these three variables.

To examine associations of social acceptability and prevalence misperceptions with being at-risk for e-cigarette use, we conducted a series of multi-level logistic regression models, with school treated as a random effect to account for differences including the percent of economically disadvantaged students. Separate logistic regression models were used with prevalence misperceptions as a predictor of at-risk status and with social acceptability as a predictor of at-risk status. We used regression models for both the full sample and for each sex, controlling for sex (for the full sample model), race/ethnicity, academic achievement, friend cigarette/e-cigarette use, and household cigarette/e-cigarette use. Also, we assessed whether there were interactions between sex and prevalence misperceptions/social acceptability in predicting at-risk status for e-cigarette use by adding interaction terms to the logistic regression models. For each logistic regression model, we used an a priori Type I error rate of 0.05 for statistical significance. We reported adjusted prevalence odds ratios (aOR), 95 % confidence intervals, and *p*-values. All analyses were performed using Stata 18.0 (College Station, Texas).

## 3 Results

### 3.1 Descriptive characteristics

For the 2020–2021 school year, the percentage of economically disadvantaged students was 70.8 %, 42.3 %, and 77.4 % for the three districts. Individual middle schools had percentages of economically disadvantaged students ranging from one-third to over ninety percent (data not shown in tables). In our study sample, a slight majority of participants (56.98 %) were Hispanic, followed by non-Hispanic White (21.32 %) and non-Hispanic Black (11.72 %) (Table 1). The majority of students reported earning mostly As (42.34 %) or mostly Bs (47.38 %) in their courses. Our study sample had 28.10 % of students report that a member of their household smokes or vapes, and 7.95 % report that a friend smokes or vapes.

**Table 1:** Descriptive characteristics of baseline sample of CATCH my breath study (n=1032 6th grade students, n=22 schools).

		**Total Sample**		**At-Risk**^a^		**Prevalence Misperceptions**^b^				**Social Acceptability**^c^				
		n	%	%	χ^2^_(df)_*p-value*	Low (%)	Medium (%)	High (%)	χ^2^_(df)_*p-value*	Low (%)	Medium (%)	High (%)	χ^2^_(df)_*p-value*	
**Total**		1032	100	36.43		50.97	23.64	25.39		27.23	29.75	43.02		
**Sex**														
	Female	542	52.52	35.06	χ^2^_(1)_ = 0.94	42.99	26.75	30.26	χ^2^_(1)_ = 29.60	26.75	28.04	45.20	χ^2^_(1)_ = 2.47	
	Male	490	47.48	37.96	*p* = 0.333	59.80	20.20	20.00	*p* = 0.000	27.76	31.63	40.61	*p* = 0.291	
**Race**^d^														
	Black	103	11.72	40.78	χ^2^_(3)_ = 1.70	42.72	30.10	27.18	χ^2^_(3)_ = 22.69	19.42	32.04	48.54	χ^2^_(3)_ = 14.79	
	Hispanic	588	56.98	36.05	*p* = 0.637	48.98	22.28	28.74	*p* = 0.001	25.34	32.82	41.84	*p* = 0.022	
	Non-Hispanic White	220	21.32	34.09		60.45	25.45	14.09		32.27	22.73	45.00		
	Other	121	11.72	38.84		50.41	21.49	28.10		33.88	25.62	40.50		
**Academic Achievement**														
	Mostly As	437	42.34	30.21	χ^2^_(2)_ = 13.31	53.32	24.49	22.20	χ^2^_(2)_ = 6.17	30.89	27.46	41.65	χ^2^_(2)_ = 7.61	
	Mostly Bs	489	47.38	40.29	*p* = 0.001	48.88	24.13	26.99	*p* = 0.187	24.13	32.72	43.15	*p* = 0.107	
	Mostly Cs-Fs	106	10.27	44.34		50.94	17.92	31.13		26.42	25.47	48.11		
**Household Cigarette/E-Cigarette Use**														
	No	742	71.90	28.98	χ^2^_(1)_ = 63.42	54.72	23.72	21.56	χ^2^_(1)_ = 22.49	29.25	30.86	39.89	χ^2^_(1)_ = 11.06	
	Yes	140	28.10	55.52	*p* = 0.000	41.38	23.45	35.17	*p* = 0.000	22.07	26.90	51.03	*p* = 0.004	
**Friend Cigarette/E-Cigarette Use**														
	No	950	92.05	34.00	χ^2^_(1)_ = 30.59	53.16	24.53	22.32	χ^2^_(1)_ = 59.62	28.74	30.74	40.53	χ^2^_(1)_ = 31.24	
	Yes	82	7.95	64.63	*p* = 0.000	25.61	13.41	60.98	*p* = 0.000	9.76	18.29	71.95	*p* = 0.000	

a Susceptible never users and experimental ever users were classified as at-risk for becoming e-cigarette users, while non-susceptible never users were classified as not at-risk

b Students were asked how much they agreed with the following statements: (1) “Most students in my middle school use e-cigarettes”; (2) “Most students my age use e-cigarettes”; and (3) “Most kids in high school use e-cigarettes.” Possible responses ranged from strongly disagree (coded as 1) to strongly agree (coded as 4) and were summed and divided into tertiles.

c Students were asked the following questions: (1) “How important is it to my parents that I do not use e-cigarettes?”; (2) “How important is it to my friends that I do not use e-cigarettes?”; (3) “How important is it to most people my age that I do not use e-cigarettes?”; (4) “How important is it to my teachers that I do not use e-cigarettes?” Possible responses ranged from strongly disagree (coded as 1) to strongly agree (coded as 4) and were summed and divided into tertiles

d For race/ethnicity, Hispanic ethnicity does not consider participant response to question of racial category. Non-Hispanic “Other” reflects those who identified as American Indian/Alaskan Native, Asian, Native Hawaiian/Other Pacific Islander, or Multi-Racial, but did not identify as Hispanic/Latino

As shown in Tables 1, 36.43% of students were at-risk for becoming e-cigarette users, with 3.68 % being ever users and 32.75 % being susceptible never users. At-risk status did not significantly vary based on sex or race. At-risk status did vary based on academic achievement (χ^2^_(2)_ = 13.31; *p*=0.001), with the proportion of students classified as at-risk being higher for those who received lower grades in school. The proportion of students classified as at-risk was also significantly higher for those reporting household cigarette/e-cigarette use (χ^2^_(1)_ = 63.42; *p*<0.001) or friend cigarette/e-cigarette use (χ^2^_(1)_ = 30.59; *p*<0.001).

About half of the total sample perceived e-cigarette use to have a low prevalence among their peers, while 24 % perceived it to have a medium prevalence, and 25 % perceived it to have a high prevalence. A higher proportion of female (χ^2^_(1)_ = 29.60; *p*<0.001) and Black, Hispanic, and “Other” racial group (χ^2^_(3)_ = 22.69; *p*=0.001) students had high prevalence misperceptions. Students with friends or family members who smoke or vape more frequently had high prevalence misperceptions (χ^2^_(1)_ = 59.62; *p*<0.001).

Approximately 27 % of students believed using e-cigarettes is not socially acceptable, 30 % reported a medium level of acceptability, and 43 % believed using e-cigarettes is socially acceptable. While social acceptability did not differ based on sex, there were statistically significant differences across race/ethnicity, with the highest percentage of Black students (49 %) reporting high social acceptability, followed by non-Hispanic white (45 %), Hispanic, and other racial/ethnic group students (χ^2^_(3)_ = 14.79; *p*=0.022).

Both prevalence misperceptions and social acceptability did not significantly vary based on academic achievement.

### 3.2 Analytic statistics

**Fig. 1:**
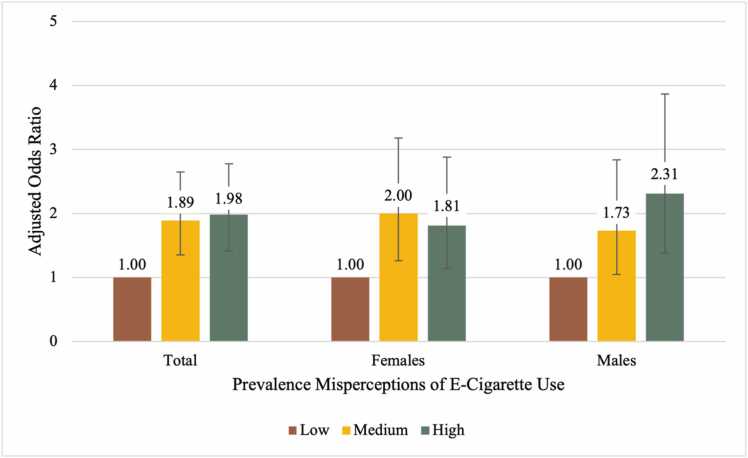
Adjusted Prevalence Odds Ratios for the Association Between Prevalence Misperceptions of E-Cigarette Use and At-Risk Status for E-Cigarette Use Among 6th Graders in Texas, CATCH My Breath Study Baseline Data, Spring 2021 (n=1032).

As presented in Table 2, students who perceived a medium and high prevalence of e-cigarette use were aOR=1.89 (95 % CI=1.35, 2.65) and aOR=1.98 (95 % CI=1.41, 2.78) times as likely to be at-risk for becoming an e-cigarette user, respectively (*p*<0.001 for both), adjusting for all covariates. As shown in Fig. 1, the relationship between prevalence misperceptions and at-risk status remained significant when stratified by sex, though females with medium misperceptions of the prevalence of e-cigarette use had slightly higher odds of being at-risk than females with high misperceptions (medium aOR=2.00; 95 % CI=1.26, 3.18; high aOR=1.81; 95 % CI=1.14, 2.88).

**Table 2:** Association between prevalence misperceptions and e-cigarette use/susceptibility among 6th graders in Texas; CATCH my breath study, 2021 Baseline Data.

		Full Sample N=1032	Females N=542	Males N=490
		Adjusted Prevalence Odds Ratio (95 % Confidence Interval)	Adjusted Prevalence Odds Ratio (95 % Confidence Interval)	Adjusted Prevalence Odds Ratio (95 % Confidence Interval)
**Prevalence Misperceptions**^a^				
	Low	1.00 (Referent)	1.00 (Referent)	1.00 (Referent)
	Medium	**1.89**⁎⁎⁎**(1.35, 2.65)**	**2.00**⁎⁎**(1.26, 3.18)**	**1.73**⁎**(1.05, 2.84)**
	High	**1.98**⁎⁎⁎**(1.41, 2.78)**	**1.81**⁎**(1.14, 2.88)**	**2.31**⁎⁎**(1.38, 3.87)**
**Sex**				
	Female	1.00 (Referent)	N/A	N/A
	Male	**1.41**⁎**(1.06, 1.86)**	N/A	N/A
**Race/Ethnicity**^b^				
	Non-Hispanic, White	1.00 (Referent)	1.00 (Referent)	1.00 (Referent)
	Hispanic/Latino	1.06 (0.74, 1.51)	0.82 (0.51, 1.32)	1.45 (0.84, 2.49)
	Non-Hispanic, Black	1.29 (0.76, 2.17)	1.22 (0.60, 2.47)	1.47 (0.67, 3.22)
	Non-Hispanic, Other	1.12 (0.68, 1.85)	0.87 (0.45, 1.69)	1.64 (0.76, 3.54)
**Academic Achievement**				
	Mostly As	1.00 (Referent)	1.00 (Referent)	1.00 (Referent)
	Mostly Bs	**1.38**⁎**(1.03, 1.85)**	**1.70**⁎**(1.14, 2.55)**	1.07 (0.70, 1.64)
	Mostly Cs-Ds	**1.70**⁎**(1.07, 2.72)**	1.95 (0.98, 3.91)	1.41 (0.73, 2.70)
**Friend Cigarette/E-Cigarette Use**				
	No	1.00 (Referent)	1.00 (Referent)	1.00 (Referent)
	Yes	**2.74**⁎⁎⁎**(1.64, 4.59)**	**2.91**⁎⁎**(1.54, 5.51)**	**2.51**⁎**(1.03, 6.13)**
**Household Cigarette/E-Cigarette Use**				
	No	1.00 (Referent)	1.00 (Referent)	1.00 (Referent)
	Yes	**2.71**⁎⁎⁎**(2.01, 3.65)**	**2.34**⁎⁎⁎**(1.57, 3.49)**	**3.33**⁎⁎⁎**(2.11, 5.24)**

⁎ *p* <0.05

⁎⁎ *p* < 0.01;

⁎⁎⁎ *p*<0.001;

a Students were asked how much they agreed with the following statements: (1) “Most students in my middle school use e-cigarettes”; (2) “Most students my age use e-cigarettes”; and (3) “Most kids in high school use e-cigarettes.” Possible responses ranged from strongly disagree (coded as 1) to strongly agree (coded as 4) and were summed and divided into tertiles.

b For race/ethnicity, Hispanic ethnicity does not consider participant response to the question of racial category. Non-Hispanic “Other” reflects those who identified as American Indian/Alaskan Native, Asian, Native Hawaiian/Other Pacific Islander, or Multi-Racial, but did not identify as Hispanic/Latino

**Fig. 2:**
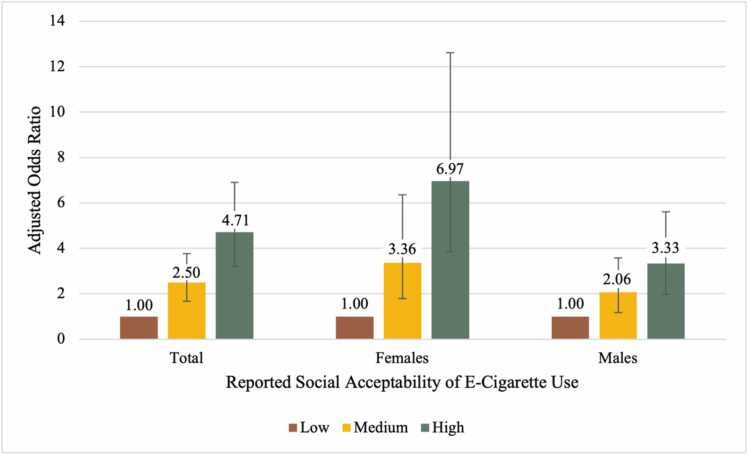
Adjusted Prevalence Odds Ratios for the Association Between Reported Social Acceptability and At-Risk Status for E-Cigarette Use Among 6th Graders in Texas, CATCH My Breath Study Baseline Data, Spring 2021 (n=1032).

As shown in Table 3, students who perceived e-cigarette use to have a medium or high level of social acceptability were aOR=2.50 (95 % CI=1.66, 3.76) and aOR=4.71 (95 % CI=3.21, 6.90) times as likely to be at-risk for becoming an e-cigarette user, respectively (*p*<0.001 for both), adjusting for covariates. In comparing across sex, medium or high social acceptability was more strongly associated with risk of e-cigarette use for females (medium aOR=3.36; 95 % CI=1.78, 6.35; *p*<0.001; high aOR=6.97; 95 % CI=3.85, 12.62; *p*<0.001) than males (medium aOR=2.06; 95 % CI=1.18, 3.58; *p*<0.05; high aOR=3.33; 95 % CI=1.97, 5.61; *p*<0.001) (Table 3 and Fig. 2).

**Table 3:** Associations between social acceptability and e-cigarette use/susceptibility among 6th graders in Texas; CATCH my breath study, 2021 Baseline Data.

		Full Sample N=1032	Females N=542	Males N=490
		Adjusted Prevalence Odds Ratio (95 % Confidence Interval)	Adjusted Prevalence Odds Ratio (95 % Confidence Interval)	Adjusted Prevalence Odds Ratio (95 % Confidence Interval)
**Social Acceptability**^a^				
	Low	1.00 (Referent)	1.00 (Referent)	1.00 (Referent)
	Medium	**2.50**⁎⁎⁎**(1.66, 3.76)**	**3.36**⁎⁎⁎**(1.78, 6.35)**	**2.06**⁎**(1.18, 3.58)**
	High	**4.71**⁎⁎⁎**(3.21, 6.90)**	**6.97**⁎⁎⁎**(3.85, 12.62)**	**3.33**⁎⁎⁎**(1.97, 5.61)**
**Sex**				
	Female	1.00 (Referent)	N/A	N/A
	Male	**1.33**⁎**(1.01, 1.77)**	N/A	N/A
**Race/Ethnicity**^b^				
	Non-Hispanic, White	1.00 (Referent)	1.00 (Referent)	1.00 (Referent)
	Hispanic/Latino	1.11 (0.77, 1.59)	0.89 (0.54, 1.46)	1.50 (0.87, 2.58)
	Non-Hispanic, Black	1.32 (0.77, 2.24)	1.19 (0.57, 2.46)	1.54 (0.70, 3.40)
	Non-Hispanic, Other	1.27 (0.76, 2.12)	1.01 (0.50, 2.01)	1.77 (0.82, 3.85)
**Academic Achievement**				
	Mostly As	1.00 (Referent)	1.00 (Referent)	1.00 (Referent)
	Mostly Bs	**1.37**⁎**(1.02, 1.85)**	**1.63**⁎**(1.07, 2.48)**	1.11 (0.72, 1.71)
	Mostly Cs-Ds	**1.70**⁎**(1.05, 2.74)**	1.58 (0.77, 3.23)	1.66 (0.87, 3.18)
**Friend Cigarette/E-Cigarette Use**				
	No	1.00 (Referent)	1.00 (Referent)	1.00 (Referent)
	Yes	**2.41**⁎⁎**(1.43, 4.04)**	**2.37**⁎**(1.23, 4.57)**	**2.47**⁎**(1.03, 5.91)**
**Household Cigarette/E-Cigarette Use**				
	No	1.00 (Referent)	1.00 (Referent)	1.00 (Referent)
	Yes	**2.78**⁎⁎⁎**(2.05, 3.78)**	**2.36**⁎⁎⁎**(1.55, 3.57)**	**3.45**⁎⁎⁎**(2.17, 5.47)**

⁎ *p* <0.05

⁎⁎ *p* < 0.01;

⁎⁎⁎ *p*<0.001;

a Students were asked the following questions: (1) “How important is it to my parents that I do not use e-cigarettes?”; (2) “How important is it to my friends that I do not use e-cigarettes?”; (3) “How important is it to most people my age that I do not use e-cigarettes?”; (4) “How important is it to my teachers that I do not use e-cigarettes?” Possible responses ranged from strongly disagree (coded as 1) to strongly agree (coded as 4) and were summed and divided into tertiles

b For race/ethnicity, Hispanic ethnicity does not consider participant response to the question of racial category. Non-Hispanic “Other” reflects those who identified as American Indian/Alaskan Native, Asian, Native Hawaiian/Other Pacific Islander, or Multi-Racial, but did not identify as Hispanic/Latino

In the interaction models, there was no significant interaction between sex and medium (aOR=0.61; 95 % CI=0.264, 1.40; *p*=0.244) or high (aOR=0.47; 95 % CI=0.22, 1.04; *p*=0.06) social acceptability compared to low social acceptability. Similarly, there was no significant interaction between sex and medium (aOR=0.86; 95 % CI=0.0.44, 1.69; *p*=0.67) or high (aOR=1.35; 95 % CI=0.69, 2.64; *p*=0.38) prevalence misperceptions compared to low prevalence misperceptions.

## 4 Discussion

In this study of Texas middle school students, sixth-grade students who perceived e-cigarette use to be more highly prevalent and socially acceptable were significantly more likely to be at-risk for becoming e-cigarette users. This relationship was especially strong for those who reported e-cigarette use as highly acceptable, with an almost 5-fold increase in risk. The associations between prevalence misperceptions and social acceptability and at-risk status for e-cigarette use remained significant when stratified by sex. These findings are consistent with past literature (Carey et al., 2019, East et al., 2019, Agaku et al., 2019, Jiang et al., 2019) and contribute to this literature base by analyzing recent data from a younger age group and exploring potential sex differences. This study contributes to a limited evidence base on the associations of prevalence misperceptions and social acceptability with at-risk status for e-cigarette use (Carey et al., 2019, Doxbeck and Osberg, 2021, East et al., 2019, Agaku et al., 2019, Jiang et al., 2019, Noland et al., 2016, Rath et al., 2021, Yong et al., 2023). To the best of our knowledge, it is among the first to quantify this relationship among middle school students in the United States during the “vaping epidemic” and explore potential sex differences.

Given that social norms are an important intervention target (Prentice, 2018), our findings can help inform the development of intervention strategies for preventing e-cigarette use. With nearly half of our sample reporting medium or high prevalence misperceptions and almost three-quarters reporting medium or high social acceptability, targeting these norms has the potential to have long-term positive health benefits for a large proportion of students. This finding takes on greater importance given that perceptions of social norms can be targeted even with limited time and resources, especially compared to factors such as friend and household e-cigarette use. It appears that intervention efforts should not only focus on the behaviors and approval of their peers, as in past research (East et al., 2019), but also on the approval of their teachers and family members. As such, our study highlights the need to include prevalence misperceptions and social acceptability in e-cigarette prevention efforts, from classroom-wide programs to mass communication campaigns. Because these social norms were associated with being susceptible to or experimenting with e-cigarettes, targeting these factors has the potential to prevent these students from transitioning to current users (Bold et al., 2018).

Further, our stratified analyses suggest that there may be sex differences in the relationship between social norms and e-cigarette use. Our descriptive analyses suggest that females more frequently overestimated the prevalence of e-cigarette use and perceived higher social acceptability than their male counterparts. Our stratified logistic regression models found a substantially larger association between social acceptability and at-risk status for females than males. When we further assessed this relationship by adding an interaction term to the overall regression model, we did not find a significant interaction between sex and these social-cognitive constructs in predicting at-risk status for e-cigarette use. However, this study was not powered to detect these differences and the sample sizes become small when stratified by both social norms tertiles and sex. Because the interaction between high social acceptability and sex approached significance, it is possible that an interaction would be detected with a larger study sample. Future research will be needed to explore possible sex differences in perceptions and e-cigarette use among youth.

There are limitations to this study that should be recognized. First, the cross-sectional nature of this study prevents us from drawing conclusions about causation. Longitudinal studies are needed to determine if the relationship between social norms and at-risk status for e-cigarette use is causal. Longitudinal data would also allow for mediation models assessing the mutual influence of prevalence misperceptions and social acceptability, as combining the two variables into one regression model in our study would have caused multicollinearity. Second, there may be response bias in the data due to the reliance on self-report surveys. Third, this study is limited in its generalizability due to the use of a convenience sample and the COVID-19 pandemic. As this was a secondary analysis of data from 6th-grade students in Texas, caution should be taken in generalizing these findings to students to other demographics. Additionally, data were collected when some students were still not attending school fully in-person due to the pandemic, which may have led to selection bias and affected students’ access to e-cigarettes. COVID-19 could have also affected students’ perceptions and behaviors related to e-cigarette use. Follow-up studies are needed to explore these relationships outside of the pandemic context. Lastly, some categories of variables had low sample sizes, as the multi-level logistic regression analyses were not adequately powered. Studies with larger sample sizes would allow for further exploration of sex differences and stratification of ever users and susceptible never users, who may have different perceptions.

## 5 Conclusions

Findings from this study indicate that medium or high prevalence misperceptions were associated with a nearly two-fold increase in odds of at-risk status, while medium and high reported social acceptability was associated with 2.5- and 4.7-fold increase in odds of at-risk status, respectively. These findings provide the foundation for targeting social norms in e-cigarette prevention efforts. Future research should assess the relationship between social norms and e-cigarette use using longitudinal data and larger sample sizes to help determine causality. As our findings contribute to a growing evidence base for the association between perceived social norms and e-cigarette use (Carey et al., 2019, East et al., 2019, Agaku et al., 2019, Jiang et al., 2019), there is a need for more research on intervention strategies to change perceived social norms related to e-cigarette use. Further exploration of sex/gender differences is also needed to understand how prevention efforts may need to be tailored to different subgroups.

## Role of funding source

Funding for this study was made possible by the National Cancer Institute (10.13039/100000054NCI) at the National Institutes of Health (10.13039/100000002NIH) grant #R01CA242171. The NCI/NIH had no role in the study design; collection, analysis or interpretation of the data; writing the manuscript; or the decision to submit the paper for publication.

## Contributors

All authors assisted with the development of the manuscript. Ms. Attri designed the study, conducted analyses, and wrote the original draft of the manuscript. Dr. Mantey assisted with the analyses and development of the manuscript. Dr. Kelder provided supervision and helped edit the manuscript. Dr. Chen assisted with statistical analyses and edited the manuscript. Dr. Springer assisted with conceptualization and edited the manuscript.

## CRediT authorship contribution statement

**Dale Mantey:** Writing – review & editing, Writing – original draft, Supervision, Formal analysis, Conceptualization. **Baojiang Chen:** Writing – review & editing, Methodology, Investigation, Formal analysis, Data curation. **Steven Kelder:** Writing – review & editing, Resources, Project administration, Funding acquisition. **Sarina Attri:** Writing – review & editing, Writing – original draft, Visualization, Formal analysis, Conceptualization. **Andrew Springer:** Writing – review & editing, Supervision, Investigation, Conceptualization.

## Declaration of Competing Interest

Dr. Mantey was a consultant for the State of Minnesota in its case against Juul Labs and Altria. Dr. Kelder was a consultant for several school districts and municipalities in its case against Juul Labs and Altria.
